# Hidden Magnetic States Emergent Under Electric Field, In A Room Temperature Composite Magnetoelectric Multiferroic

**DOI:** 10.1038/s41598-017-13760-y

**Published:** 2017-11-13

**Authors:** J. D. Clarkson, I. Fina, Z. Q. Liu, Y. Lee, J. Kim, C. Frontera, K. Cordero, S. Wisotzki, F. Sanchez, J. Sort, S. L. Hsu, C. Ko, L. Aballe, M. Foerster, J. Wu, H. M. Christen, J. T. Heron, D. G. Schlom, S. Salahuddin, N. Kioussis, J. Fontcuberta, X. Marti, R. Ramesh

**Affiliations:** 1 Department of Materials Science and Engineering, University of California, Berkeley, California 94720 USA; 2 Institut de Ciència de Materials de Barcelona (ICMAB-CSIC), Campus UAB, Bellaterra, 08193 Barcelona, Spain; 3 Oak Ridge National Laboratory, Center for Nanophase Materials Sciences, Oak Ridge, Tennessee 37831 USA; 4 Department of Physics, California State University, Northridge, California, 91330-8268 USA; 5 Catalan Institute of Nanoscience and Nanotechnology (ICN2), CSIC and The Barcelona Institute of Science and Technology, Campus UAB, Bellaterra, 08193 Barcelona, Spain; 6 Max Planck Institute of Microstructure Physics, Weinberg 2, D-06120 Halle (Saale), Germany; 7 Departament de Física, Universitat Autònoma de Barcelona, E-08193 Bellaterra, Spain; 8 Institució Catalana de Recerca i Estudis Avançats (ICREA), Passeig Lluís Companys 23, E-08010 Barcelona, Spain; 9 Materials Sciences Division, Lawrence Berkeley National Laboratory, Berkeley, California, 94720 USA; 10 ALBA Synchrotron Light Facility, Carrer de la Llum 2-26, Cerdanyola del Vallès, Barcelona, 08290 Spain; 11 Department of Materials Science and Engineering, Cornell University, Ithaca, New York, 14850 USA; 12 Department of Materials Science and Engineering, University of Michigan, Ann Arbor, Michigan 48109 USA; 13 Department of Electrical Engineering and Computer Science, University of California, Berkeley, California, 94720 USA; 14 Department of Physics, University of California, Berkeley, California, 94720 USA; 15 Institute of Physics ASCR, v.v.i., Cukrovarnicka 10, 162 53 Praha 6, Czech Republic

## Abstract

The ability to control a magnetic phase with an electric field is of great current interest for a variety of low power electronics in which the magnetic state is used either for information storage or logic operations. Over the past several years, there has been a considerable amount of research on pathways to control the direction of magnetization with an electric field. More recently, an alternative pathway involving the change of the magnetic state (ferromagnet to antiferromagnet) has been proposed. In this paper, we demonstrate electric field control of the Anomalous Hall Transport in a metamagnetic FeRh thin film, accompanying an antiferromagnet (AFM) to ferromagnet (FM) phase transition. This approach provides us with a pathway to “hide” or “reveal” a given ferromagnetic region at zero magnetic field. By converting the AFM phase into the FM phase, the stray field, and hence sensitivity to external fields, is decreased or eliminated. Using detailed structural analyses of FeRh films of varying crystalline quality and chemical order, we relate the direct nanoscale origins of this memory effect to site disorder as well as variations of the net magnetic anisotropy of FM nuclei. Our work opens pathways toward a new generation of antiferromagnetic – ferromagnetic interactions for spintronics.

## Introduction

In recent years, the ever increasing need for large scale data storage in computing systems has kindled significant interest in new materials and new physical phenomena that could lead to denser and more energy efficient memory systems. One approach is the heat assisted magnetic recording (HAMR) for which low Neel temperature AFMs have been suggested to play a key role^1^. In this context, the AFM to FM phase transition makes FeRh a serious contender for insertion into heterostructures utilizing exchange bias. Typically, in magnetic systems, defects impede domain wall motion, increase the coercivity, lower magnetization, and decrease spin polarization. Going beyond this traditional thinking, in this work we demonstrate that it is possible to exploit crystalline defects in FeRh to induce a voltage driven phase transition in FeRh that leads to a mixed AFM/FM state. This mixed phase can then be read out through a strong magnetoresistance (MR) effect. The presence of the mixed phase hints to a potential source of rich physical phenomena and the ability to control and probe the transition electrically. This furthermore suggests that an electrically assisted magnetic recording (EAMR) is achievable in a single magnetic material.

We employ a thin layer of Fe_0.50_Rh_0.50_ (FeRh) which is an intermetallic compound that exhibits an antiferromagnetic (AFM) to ferromagnetic (FM) phase transition that can be controlled by strain, heat, or magnetic field^2–9^. Concurrent with this metamagnetic phase transition, FeRh displays a very large change in resistivity (~30–40%)^2^ which can be triggered by any of the aforementioned stimuli. Our device is comprised of a piezoelectric substrate (for example, (0.72)PbMg_1/3_Nb_2/3_O_3_ - (0.28)PbTiO_3_ (PMN-PT) or BaTiO_3_(BTO)) which allows the tuning of the strain exerted on the magnetic material. This combination of materials has achieved, in previous work, an electrically driven modulation of magnetization^6,9,10^ by as large as 70 emu/cm^3^ (~21% isothermal magnetization modulation)^6^ and resistivity modulation of 8–22%^7,8^ thus proving the magnetoelectric coupling of the heterostructure. Specific details for the very same samples employed in this work are found elsewhere^7,8^.

**Figure 1:**
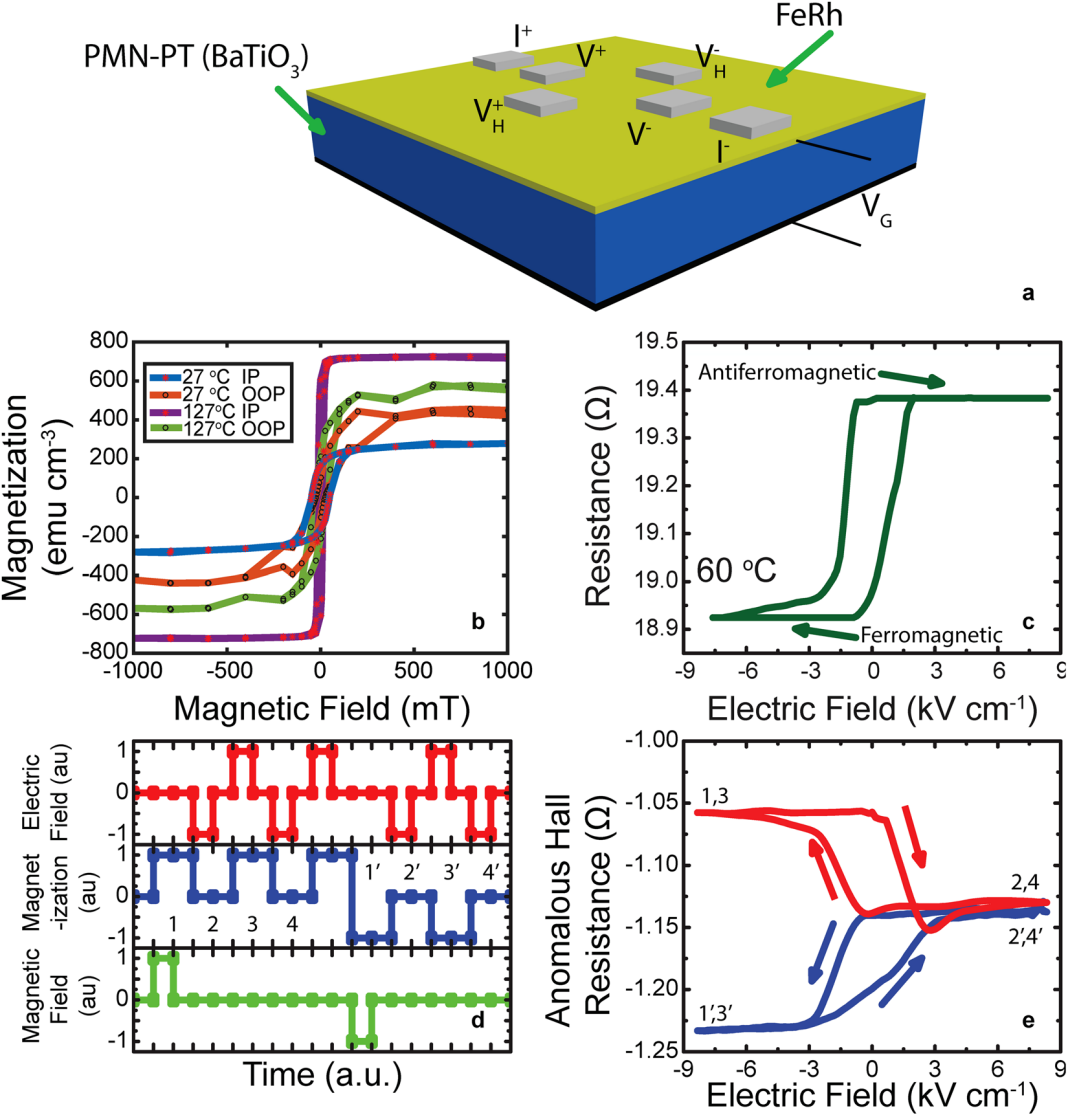
FeRh Magnetic Anisotropy and Electric Field Control (**a**) Transport device geometry. (**b**) In-plane and out-of-plane magnetization hysteresis loops measured by Superconducting Quantum Interference Device (SQUID) at 27 °C and 127 °C. (**c**) Electric field dependence of the longitudinal resistance of FeRh (30 nm)/PMN-PT. (**d**) A step-wise illustration of how the memory element is programmed into a magnetic state (step 1 or 1′), “cloaked” by the application of an electric field (step 2 or 2′) and “uncloaked” by the application of an opposite polarity electric field (step 3 or 3′) of our proposed cloaked magnetic memory device functions. (**e**) Electric-field dependences of the anomalous Hall resistance (AHR) for the two magnetic states.

Our model system consists of a 30 nm thick FeRh layer that is deposited by ultrahigh vacuum sputtering onto a single crystal of PMN-PT at a deposition temperature of 350 °C. To demonstrate the generality of this approach, a single crystal BaTiO_3_ was also used as a piezoelectric substrate. Prior to our magnetoelectric studies, we characterized the crystalline quality of the FeRh layer (a sampling is shown in Supplementary Figure S1). 4-probe transport measurements were carried out as a function of temperature and electric field, using a test structure shown schematically in Fig. 1(a). Armed with this baseline, we now present results of temperature and electric field dependent magnetic and magnetotransport studies. Figure 1(b) presents temperature dependent SQUID magnetic hysteresis loops for the FeRh, measured in-plane and out-of-plane at 27 °C and 127 °C (more detailed magnetic data is presented in Supplemental Figure S2 for the PMNPT substrate and in Figure S3 for the BaTiO_3_ substrate). Upon lowering the temperature, two changes occur. First, the absolute magnetic moment is reduced, consistent with the emergence of the antiferromagnetic phase. Second, and perhaps more importantly, the magnetization is now directed significantly more out-of-plane, as illustrated in the comparison of the in-plane versus out-of-plane magnetic hysteresis loops. This out-of-plane component of the magnetization is key to the detectability of this state (in our case through anomalous Hall resistance measurements^11,12^ [AHR, R_xy_], described below).

In the mixed AFM-FM state (for example at a temperature of 60 °C) application of an out-of-plane electric field to the heterostructure leads to a strong change in the (longitudinal) resistance, Fig. 1(c) (for simplicity, the corresponding data for the BaTiO_3_ substrate is shown in Supplemental Figure S4), which is directly attributed to the electric field control of the magnetic state (FM vs. AFM)^6–8^. Positive (negative) electric fields produce an increase (decrease) of electrical resistance, corresponding to an increase in the AFM (FM) phase fraction due to the strain-induced preference for this phase^6–8^. The temperature dependence of the longitudinal and transverse (i.e., Anomalous Hall) resistances for the two types of substrates are shown in Supplemental Figure S5. Using this as the starting point, we now describe how such a heterostructure can be used to cloak the written magnetic state. The strategy we propose is schematically summarized in Fig. 1(d). The memory element is programmed into the UP and DOWN states, [step 1 or 1′] with a magnetic field; a subsequent electric field [step 2 or 2′] converts it into the AFM phase thus cloaking and protecting it from external perturbation. An electric field of opposite polarity [step 3 or 3′] converts it back to the original FM state. The main experimental result to validate this protocol is summarized in Fig. 1(e) which shows the AHR of the FeRh layer as a function of the electric field applied to the underlying piezoelectric. After magnetizing the FeRh into the UP (or DOWN) states, a negative electric field, favors ferromagnetism (state 1 or 1′), while a positive field converts it into the AFM state with a lower AHR (state 2 or 2′), which are essentially the same.

In order to understand the effects of in-plane strain and the relative volume fraction of the FM phase on the magnetic moment orientation, we have carried out *ab-initio* electronic structure calculations of the volume-averaged magnetic anisotropy energy (MAE), ${\bar{K}}_{V}(\varepsilon ,x)=(1-x){K}_{V}^{AFM}(\varepsilon )+x\,{K}_{V}^{FM}(\varepsilon )$. Here, $\varepsilon =(a-{a}_{o}^{FM})/{a}_{o}^{FM}$, is the strain relative to the equilibrium FM-FeRh lattice constant, ${a}_{o}^{FM}$, ${K}_{V}^{AFM(FM)}(\varepsilon )$ is the strain dependent intrinsic bulk MAE for the AFM (FM) FeRh phase and $x=\frac{{V}^{FM}}{{V}^{FM}+{V}^{AFM}}$ is the relative volume fraction of the FM phase. In order to investigate the effect of magnetic shape anisotropy energy (MSAE) on the FM thin film, we have also calculated the effective MAE, ${K}_{eff}^{FM}(\varepsilon )={K}^{FM}(\varepsilon )-2\pi {M}^{2}(\varepsilon ,x)$, where $M\,(\varepsilon ,x)=x({m}_{Fe}(\varepsilon )+{m}_{Rh}(\varepsilon ))/V(\varepsilon )$, ${m}_{Fe}(\varepsilon )$, ${m}_{Rh}(\varepsilon )$, and $V(\varepsilon ),$ are the weakly strain-dependent magnetic moments of the Fe and Rh atoms, and the volume of the formula unit cell, respectively.

**Figure 2:**
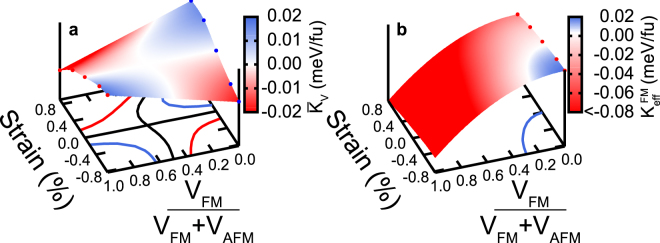
*Ab-initio* calculations results of the FeRh magnetic anisotropy as a function of strain. (**a**) The volume-averaged magnetic anisotropy energy, ${\bar{K}}_{V}$, as a function of strain and FM/AFM volume fraction. (**b**) The net magnetic anisotropy energy of the FM phase is shown, including demagnetization effects.

In Fig. 2(a) we show the variation of the volume-averaged MAE, ${\bar{K}}_{V}$ with strain and relative volume fraction of the FM phase. We find that the MAE is positive (negative) under compressive (tensile) strain for the FM phase, indicating an out-of-plane (in-plane) orientation of the magnetic moments. In sharp contrast, the strain dependence of the MAE in the AFM phase is reversed, consistent with previous results^13^. The variation of the effective MAE, ${K}_{eff}^{FM}$, of the FM phase is shown in Fig. 2(b). Interestingly, for x = 1, the MSAE renders ${K}_{eff}^{FM} < 0$ in the entire strain range, demonstrating that when the entire volume is in the FM phase, the spin orientation is in-plane. As the relative volume fraction of the FM phase decreases, the effect of MSAE also decreases and ${K}_{eff}^{FM}$ is determined primarily by ${K}_{V}^{FM}$ yielding an out-of-plane spin orientation under compressive strain.

**Figure 3:**
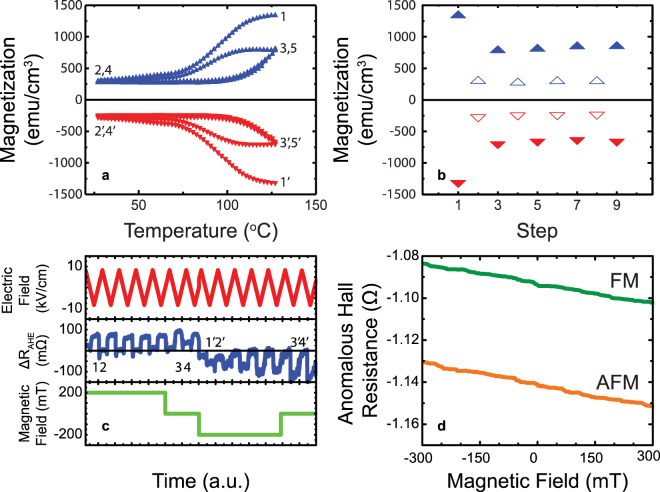
Phase Modulation and Invisible Memory Proof of Concept (**a**) Magnetization versus temperature loops measured at 0 mT, after saturating the sample (±500 mT) at 127 °C. Arrows indicate increasing or decreasing temperature. (**b**) Magnetic moment at 127 °C (solid symbols) and 27 °C (open symbols) measured after successive thermal cycling at 0 mT. (**c**) Anomalous Hall resistance as a function of time (at 60 °C) at positive (200 mT), zero, and negative (−200 mT) magnetic fields through a series of different voltage cycles. Between the 0 and −200 mT fields, a field cooling was carried out. (**d**) AHR as a function of magnetic field for the two 0 electric-field states at 25 °C. The magnetic response is due to the ordinary Hall effect, and are separated due to differing magnetization states, AFM and FM.

Supporting evidence for the electric field modulation of the magnetic state described in Fig. 1(e) comes from analogous temperature dependence of the magnetic state, as shown in Fig. 3(a), which shows the sequence of steps in which the FeRh layer is oriented in a magnetic field of +(−) 500 mT, then cooled from the FM state (state 1 or 1′) at 127 °C in to an essentially AFM state at 27 °C (state 2 or 2′). Upon heating back to 127 °C, the same ferromagnetic “polarity” is retrieved (i.e. state 3 or 3′), suggesting the existence of FM kernels within the AFM phase, which contain the directional magnetic information and assist in attaining the originally written FM state upon heating. Indeed, XMCD-PEEM studies of such films through the same thermal cycling (Supplemental Figure S6) reveal that the same ferromagnetic state is attained as the sample is heated into the ferromagnetic state, cooled into the antiferromagnetic state and then returned to the high temperature ferromagnetic state. Figure 3(b) demonstrates that such a reversal can be carried out over several cycles. The corresponding electric field cycling results are presented in Fig. 3(c) which shows the AHR as a function of time at positive, zero and negative magnetic fields as a function of electric field cycling at 60 °C. It is clear that the ability to distinguish the two magnetic states is electric field polarity dependent and stable over several cycles. The horizontal line in Fig. 3(c) indicates the AFM or “cloaked” state, while the data significantly above or below the line represent a positive or negative magnetization state. With a negative applied electric field, the magnetization direction of this “cloaked” state is revealed. To establish the robustness of this data against external magnetic fields, we deliberately probed the magnetic field dependence of the AHR in both the FM and AFM states at zero electric field, as shown in Fig. 3(d). The fact that the magnetic field dependence for the two states do not crossover implies that the AFM state is stable in an external magnetic field, at least up to 300 mT at a temperature of 27 °C.

**Figure 4:**
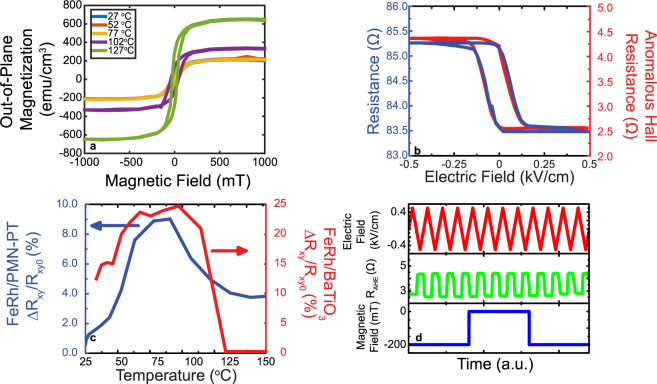
(**a**) Temperature dependence of the magnetic hysteresis; (**b**) normal and anomalous Hall resistance at 60 °C; (**c**) a comparison of the normalized anomalous Hall resistance for FeRh films grown on PMNPT and BaTiO_3_ substrates; (**d**) demonstration of the robustness of the “protected” state to external magnetic fields.

We establish the generality of this approach by carrying out the same set of experiments using a single crystal BaTiO_3_ as the substrate. Figure 4(a) shows the magnetic hysteresis loops as a function of temperature, again indicating the emergence of the AFM state from the high temperature FM state. Normal and anomalous Hall transport measurements, Fig. 4(b), show a strong electric field driven hysteresis, consistent with what was observed with PMNPT substrates. The temperature dependence of the normalized anomalous Hall resistance for the two piezoelectric substrates are compared in Fig. 4(c); the overall trend is similar, although the films on BaTiO_3_ substrates show a larger modulation and a sharper temperature dependence, reflective of the better crystalline quality. Finally, the stability of the “protected” state to external magnetic fields is demonstrated in Fig. 4(d), which is again consistent with that measured with PMNPT as the piezoelectric substrate. These results demonstrate the generality of this approach.

**Figure 5:**
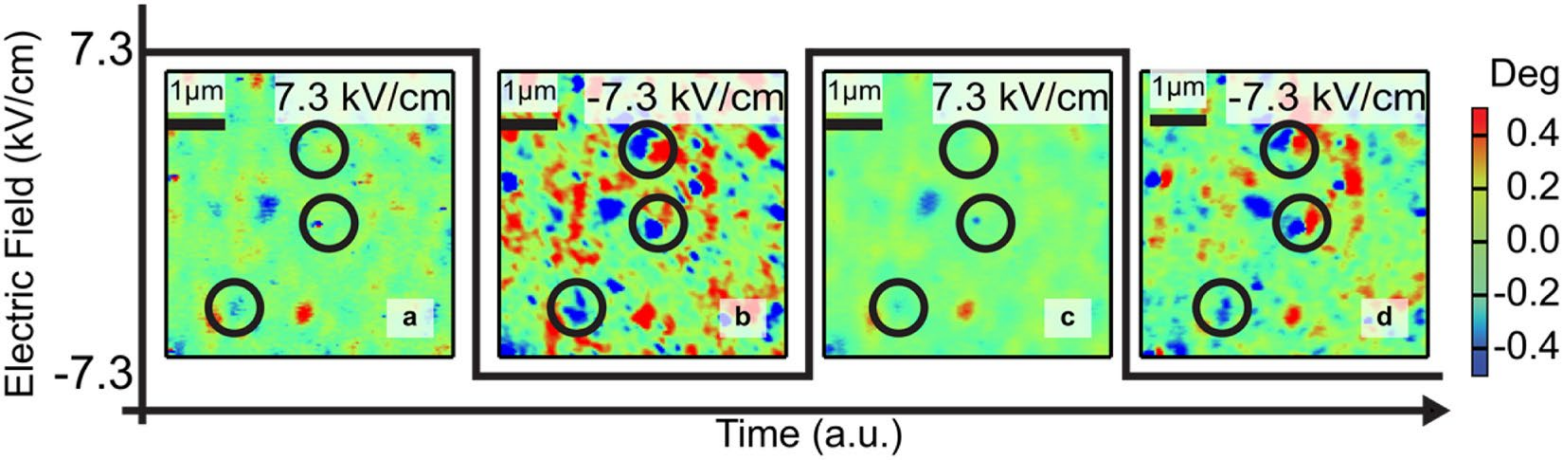
Local Memory of Directionality. Magnetic force microscopy of the FeRh layer at 60 °C under a DC electric field of ± 7.3 kV/cm is applied. Images are labeled sequentially with their corresponding electric field. The magnetic response in (**a**) and (**c**) are drastically diminished, when compared with the same region in (**b**) and (**d**). The local magnetic memory effect is observed here as the local magnetization is reversibly modified with electric field.

We now probe the microscopic details of such a directional magnetic memory, using magnetic force microscopy (MFM) as a function of applied electric field. We observed measurable changes in the image contrast upon the application of positive and negative electric fields. Figure 5(a), obtained with a positive electric field of 7.3 kV/cm shows an essentially “uniform” contrast, indicative of an essentially antiferromagnetic state (i.e., no significant magnetic moment); upon the application of a −7.3 kV/cm electric field (Fig. 5(b)), the image contrast changes with the emergence of regions with strong contrast (in blue and red), indicative of the emergence of the ferromagnetic state. It is particularly important to note that the contrast for a given location in the sample is reversibly changed from red (or blue) to green, again providing supporting evidence to the notion that a specific ferromagnetic polarity is retained in that location. The corresponding data for FeRh on BaTiO_3_ is shown in Supplemental Figure S7 and it is generally consistent with the observations in Fig. 5.

It has been reported earlier that the FM state set at high temperature can be imprinted in the AFM state at room temperature by fixing the direction of the antiferromagnetic axis by in-field magnetic cooling from the high temperature FM phase down to the low temperature AFM phase^14^. Therefore, the observed memory effect can be attributed to a reciprocal effect, in which the magnetic orientation is frozen in the AFM state and drives the magnetization orientation in the FM state upon subsequent warming (or the application of a negative electric field). The role of localized structural and antisite defects in the FeRh film structure is likely a key element for such a memory effect. Indeed, a dependence on the low temperature ferromagnetic phase fraction is observed. FeRh films deposited on single-crystalline MgO show an annealing dependence on the memory effect that is magnified for FeRh/PMN-PT. The high quality films on MgO do not show a retained magnetic moment at room temperature and, in agreement with prior work^15^, we observe no memory effect. Therefore, we conclude that the coexistence of FM/AFM phases at room-temperature is required to store the magnetic information and that the minority FM domains present at room temperature must play a crucial role in the observed memory effect. The coexistence of magnetic phases has been long exploited in the well-known magnetic shape-memory alloys that show magnetic memory while crossing the austenite to martensite phase transition at which the magnetic state changes^15,16^. It appears that a similar phenomenology is at play here, with the advantage that the room-temperature AFM phase can mask the coexisting FM nuclei. It is worth noting that, in contrast to most common situations where single crystalline substrates are a demand for optimal thin film properties, here poly-domain substrates are advantageous and thus we expect to observe similar memory effects utilizing a wide range of inexpensive substrates. Finally, exploiting memory effects such as those described here require materials that display near room temperature AFM to FM transitions. Fortunately several alloys, such as Mn_2_Sb and related compounds^17–19^ are already known.

## Methods

Thin films were prepared by DC magnetron sputtering onto PMN-PT and BaTiO_3_ substrates that were heated up to 300 °C in a base pressure of 10^−8 ^ Torr. Subsequently, Ar gas was introduced (3 mTorr) and films were grown using 50 W power at a rate of 1 nm min^−1^ using a stoichiometric FeRh target.

Magnetic characterization with magnetic fields up to 1 T was carried out in a superconducting quantum interference device by Quantum Design using the Reciprocating Sample Option.

Transport experiments utilized four-probe resistance contacts with additional transverse contacts, perpendicular to the current direction separated by 500 μm in a magnet. Longitudinal and transverse resistances are averages of the positive and negative current values. Currents were applied along the [100] directions. Electric fields were applied between a current lead and the back side of the sample.

The *ab-initio* electronic structure calculations have been carried out within the framework of the projector augmented-wave formalism^20^ as implemented in the Vienna *ab-initio* simulation package (VASP)^21,22^, with the generalized gradient approximation for the exchange-correlation functional parameterized by Perdew-Burke-Ernzerhof (PBE)^23^. The plane-wave cutoff energy is 500 eV and the tetrahedron method is used for the Brillouin zone integration with Γ centered k -mesh of 24 × 24 × 24 grid. Spin-orbit coupling is included using the second-variation method^24^ employing the scalar-relativistic eigenfunctions of the valence states. The bulk MAE per formula unit for FM (AFM) phase is determined from ${K}_{V}^{AFM(FM)}=({E}_{[100]}-{E}_{[001]})$, where ${E}_{[100]}$ and ${E}_{[001]}$ are the total energies with magnetization along the [100] and [001] directions, respectively.

Magnetic Force Microscopy images were acquired using an Asylum MFP3D AFM with a magnetic CoCr coated tip. The sample was heated to 60 °C using Asylum’s polymer heater. Voltage was applied between the grounded sample surface and the back side of the sample during measurement.

## Electronic supplementary material

Hidden Magnetic States Emergent Under Electric Field, In A Room Temperature Composite Magnetoelectric Multiferroic
