# Supplementary material for: Hidden Magnetic States Emergent Under Electric Field, In A Room Temperature Composite Magnetoelectric Multiferroic
Source: Sci Rep. 2017 Nov 13;7:15460. doi: 10.1038/s41598-017-13760-y (PMC5684172; doi:10.1038/s41598-017-13760-y)
Supplement: Supplementary file 1 — Hidden Magnetic States Emergent Under Electric Field, In A Room Temperature Composite Magnetoelectric Multiferroic [file 41598_2017_13760_MOESM1_ESM.pdf]

Hidden Magnetic States Emergent Under Electric Field, In A Room Temperature Composite Magnetoelectric Multiferroic

J.D. Clarkson[1], I. Fina[2], Z. Q. Liu[1,3], Y. Lee[1], J. Kim[4], C. Frontera[2], K. Cordero[5], S. Wisotzki[6], F. Sanchez[2], J. Sort[7,8], S. L. Hsu[1,9], C Ko[1], L. Aballe[10], M. Foerster[10], J. Wu[1,9], H.M. Christen[3], J. T. Heron[11,12], D.G. Schlom[11], S. Salahuddin[13], N. Kioussis[4], J. Fontcuberta[2], X. Marti[14] and R. Ramesh*[1,9,14]

[1] Department of Materials Science and Engineering, University of California, Berkeley, California 94720, USA
[2] Institut de Ciència de Materials de Barcelona (ICMAB-CSIC), Campus UAB, Bellaterra 08193, Barcelona, Spain
[3] Oak Ridge National Laboratory, Center for Nanophase Materials Sciences, Oak Ridge, Tennessee 37831, USA
[4] Department of Physics, California State University, Northridge, California 91330-8268, USA
[5]Catalan Institute of Nanoscience and Nanotechnology (ICN2), CSIC and The Barcelona Institute of Science and Technology, Campus UAB, Bellaterra, 08193 Barcelona, Spain
[6] Max Planck Institute of Microstructure Physics, Weinberg 2, D-06120 Halle (Saale), Germany
[7] Departament de Física, Universitat Autònoma de Barcelona, E-08193 Bellaterra, Spain
[8] Institució Catalana de Recerca i Estudis Avançats (ICREA), E-08010 Barcelona, Spain
[9] Materials Sciences Division, Lawrence Berkeley National Laboratory, Berkeley, California 94720, USA
[10]ALBA Synchrotron Light Facility, Carrer de la Llum 2-26, Cerdanyola del Vallès, Barcelona 08290, Spain
[11] Department of Materials Science and Engineering, Cornell University, Ithaca, New York 14850
[12] Department of Materials Science and Engineering, University of Michigan, Ann Arbor, Michigan 48109, USA
[13] Department of Electrical Engineering and Computer Science, University of California, Berkeley, California 94720, USA
[14] Department of Physics, University of California, Berkeley, California 94720, USA
[15] Institute of Physics ASCR, v.v.i., Cukrovarnicka 10, 162 53 Praha 6, Czech Republic

1. X-ray diffraction and TEM of FeRh deposited on PMN-PT and $BaTiO_3$ substrates.

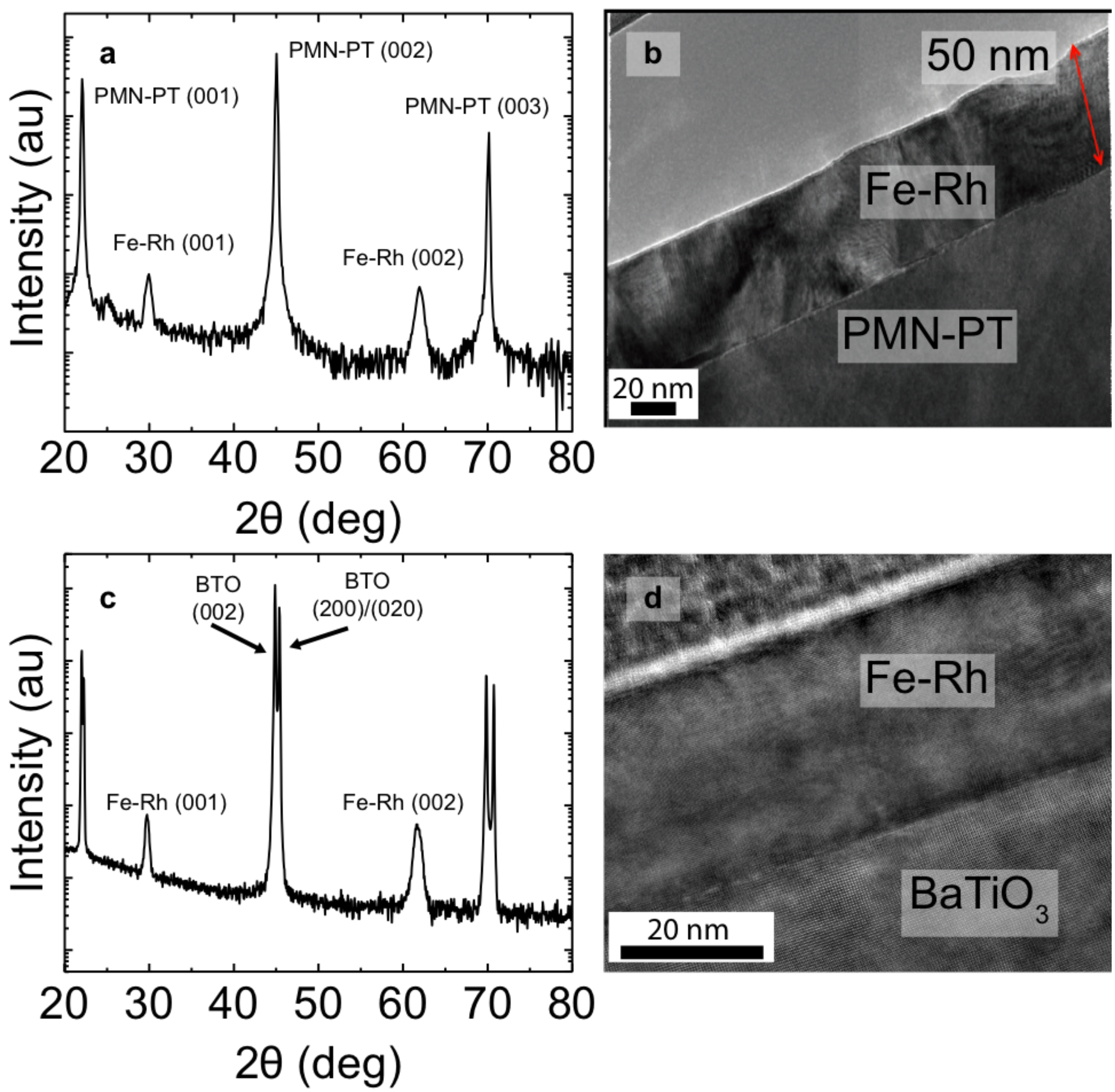

FIG. S1: (A) Room temperature out-of-plane x-ray diffraction, 2θ/θ, of 30 nm FeRh/PMN-PT exhibiting a preferred (110) out-of-plane orientation. (B) TEM of a 50 nm FeRh film on PMN-PT. The film exhibits multiple grains. (C) Room temperature out-of-plane x-ray diffraction, 2θ/θ, of FeRh/$BaTiO_3$ exhibiting a (001) out-of-plane orientation. (D) TEM of a 30 nm FeRh film on $BaTiO_3$. No grain boundaries are observed, confirming a single crystal thin film with high crystallinity. Scale bars in (B) and (D) are 20 nm.

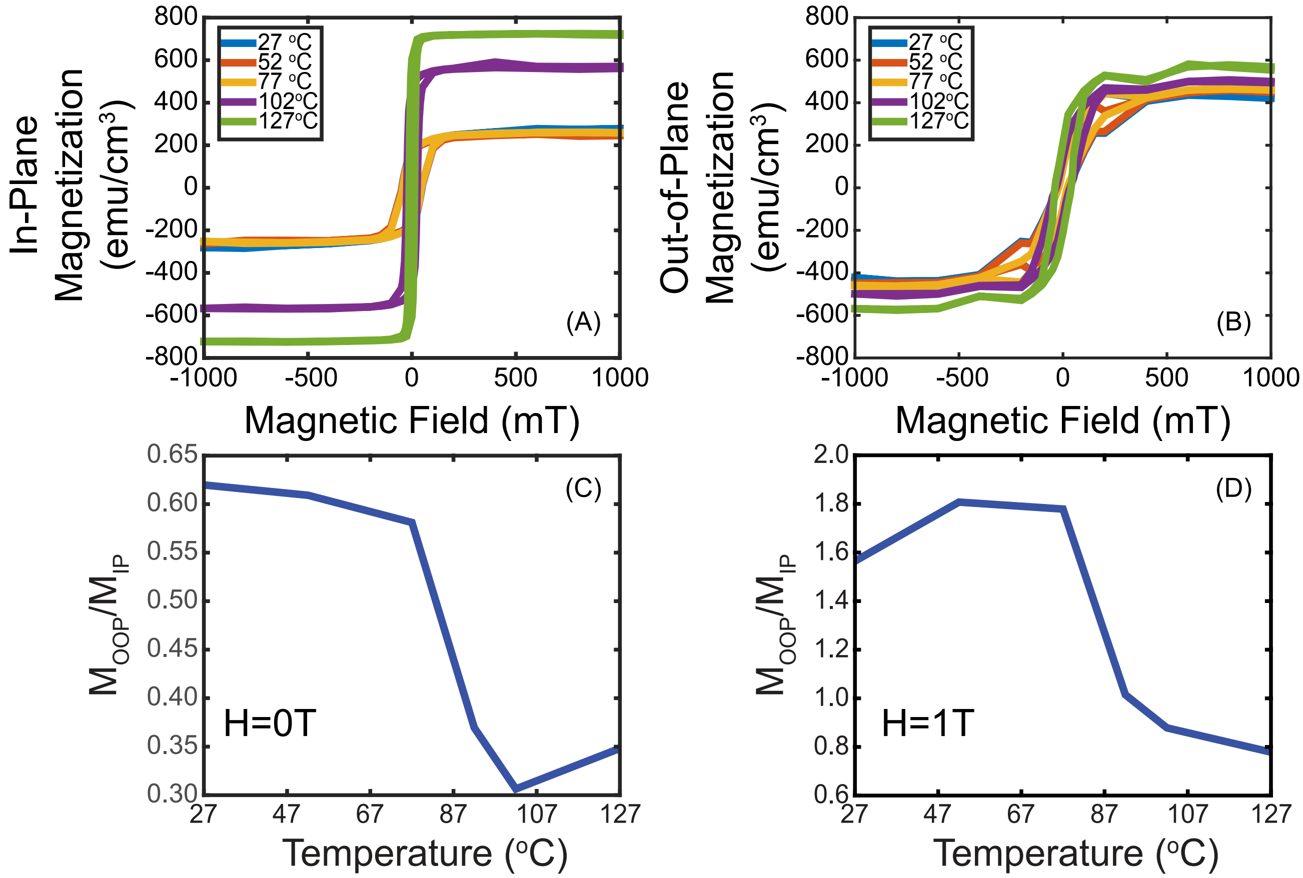

FIG. S2: (A) In-plane and (B) out-of-plane magnetization hysteresis loops at various temperatures across the magnetic phase transition for FeRh/PMN-PT. Ratios of magnetization components at (C) zero magnetic field and (D) 1 T, depicting the temperature dependence of the magnetic anisotropy for FeRh/PMN-PT. An out-of-plane magnetization direction is preferred below the transition temperature.

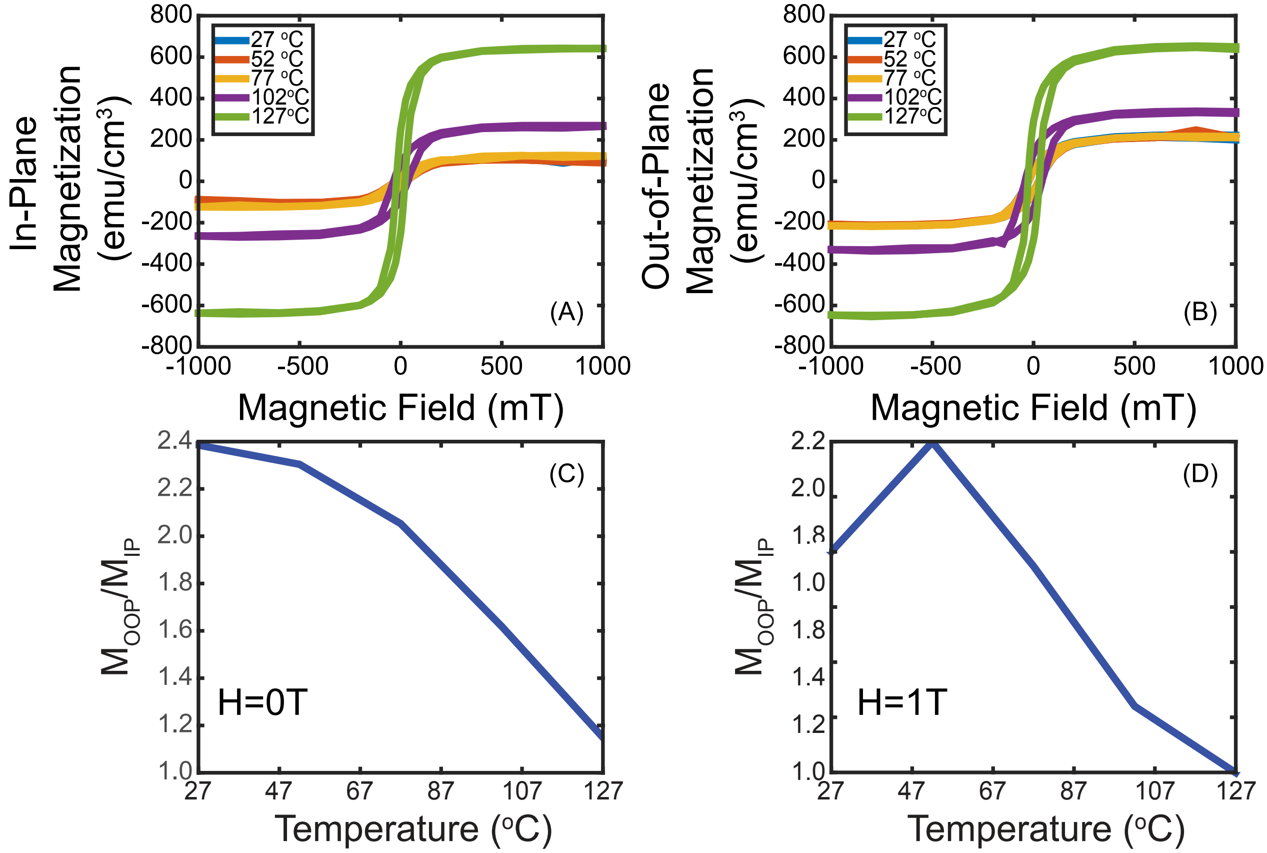

FIG. S3: (A) In-plane and (B) out-of-plane magnetization hysteresis loops at various temperatures across the magnetic phase transition for FeRh/ $BaTiO_3$. Ratios of magnetization components at (C) zero magnetic field and (D) 1 T, depicting the temperature dependence of the magnetic anisotropy as a function of temperature for FeRh/$BaTiO_3$. An out-of-plane magnetization direction is preferred below the transition temperature.

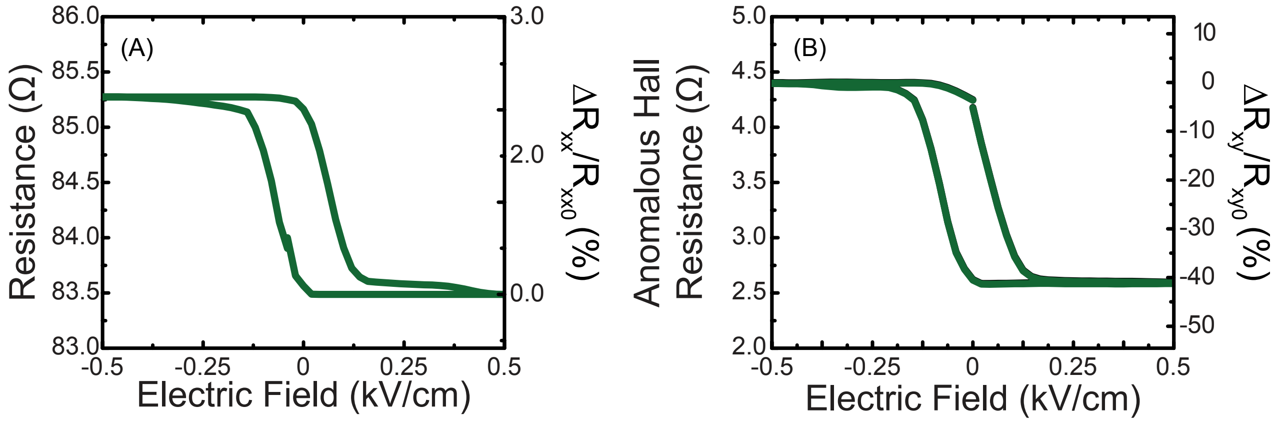

FIG. S4: Longitudinal (R_{xx}) (A) and transverse (R_{xy}, AHR) (B) resistance measurements as a function of electric field for Fe-Rh/$BaTiO_3$ at 60 °C with no external magnetic field. The percent change in the transverse resistance is more than an order of magnitude larger than the percent change in longitudinal resistance indicating that the loop in the transverse resistance is dependent on the out-of-plane magnetization.

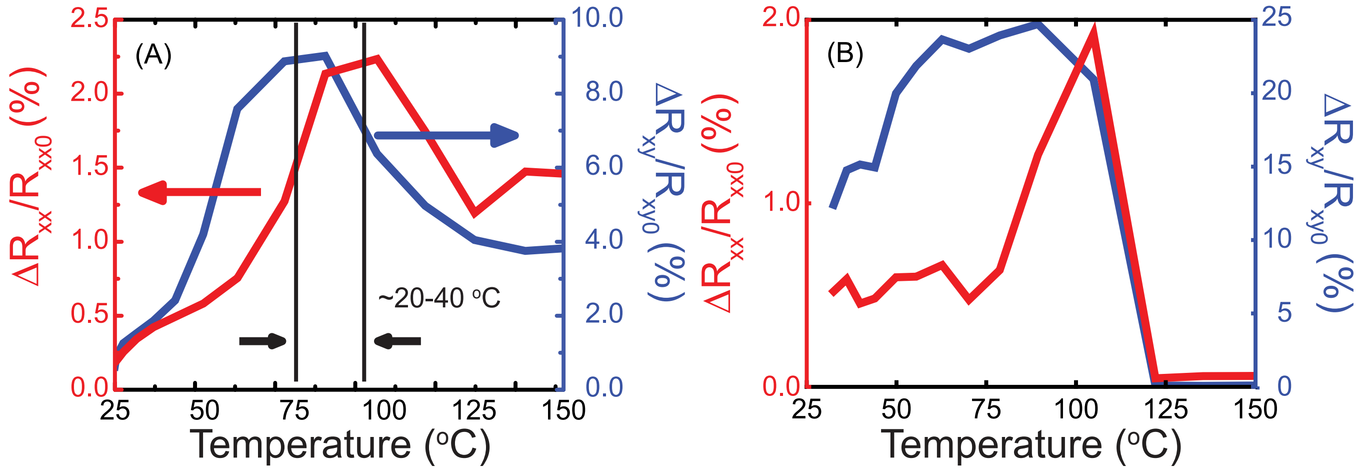

FIG. S5: Temperature dependence of electric field control of FeRh longitudinal and transverse resistances for FeRh/PMN-PT (A) and FeRh/$BaTiO_3$ (B). There is a marked shift in the transverse (AHR) modulation to lower temperature as a result of the anisotropy rotation at lower temperatures.

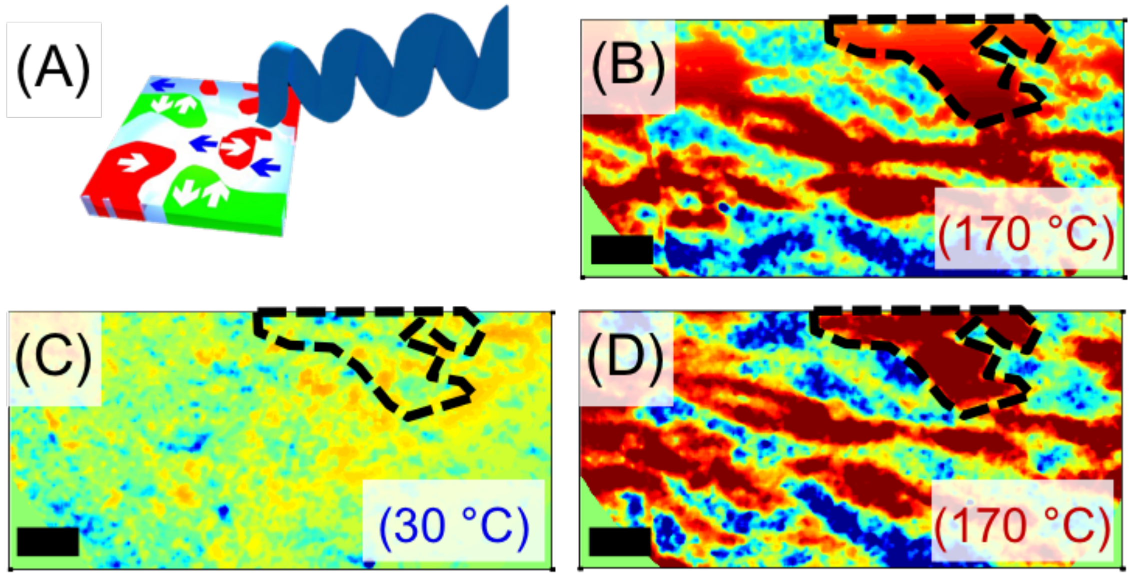

FIG. S6: (A) Sketch of the PEEM-XMCD experiment configuration. An x-ray photon (indicated by the blue wave) is directed at a FeRh sample that has a mixture of antiferromagnetic (green) and ferromagnetic (white and red) domains. (B-D) PEEM-XMCD image collected in the very same region at high temperature (170 °C), low temperature (30 °C) and high temperatures (170 °C), respectively, recorded sequentially. The image in (B) has been recorded after saturating the sample at high temperatures, cooling to room temperature in zero field cooling conditions and heating it once. Note that the three images have been collected at magnetic remanence and no magnetic field has been applied during the (B) to (D) measuring sequence. The false color scale corresponds to the projection of the magnetization onto the incident x-ray beam (horizontal from the right). Domains with magnetization parallel and antiparallel to the x-ray incidence have opposite contrast (blue and red colors). Perpendicular domains or domains with zero magnetic moment have green color. The location of the ferromagnetic phase and its magnetization orientation can be reproducibly erased and restored through a heating and cooling cycle as illustrated by the region highlighted by the black dashed line. Scale bar correspond to 1 μm.

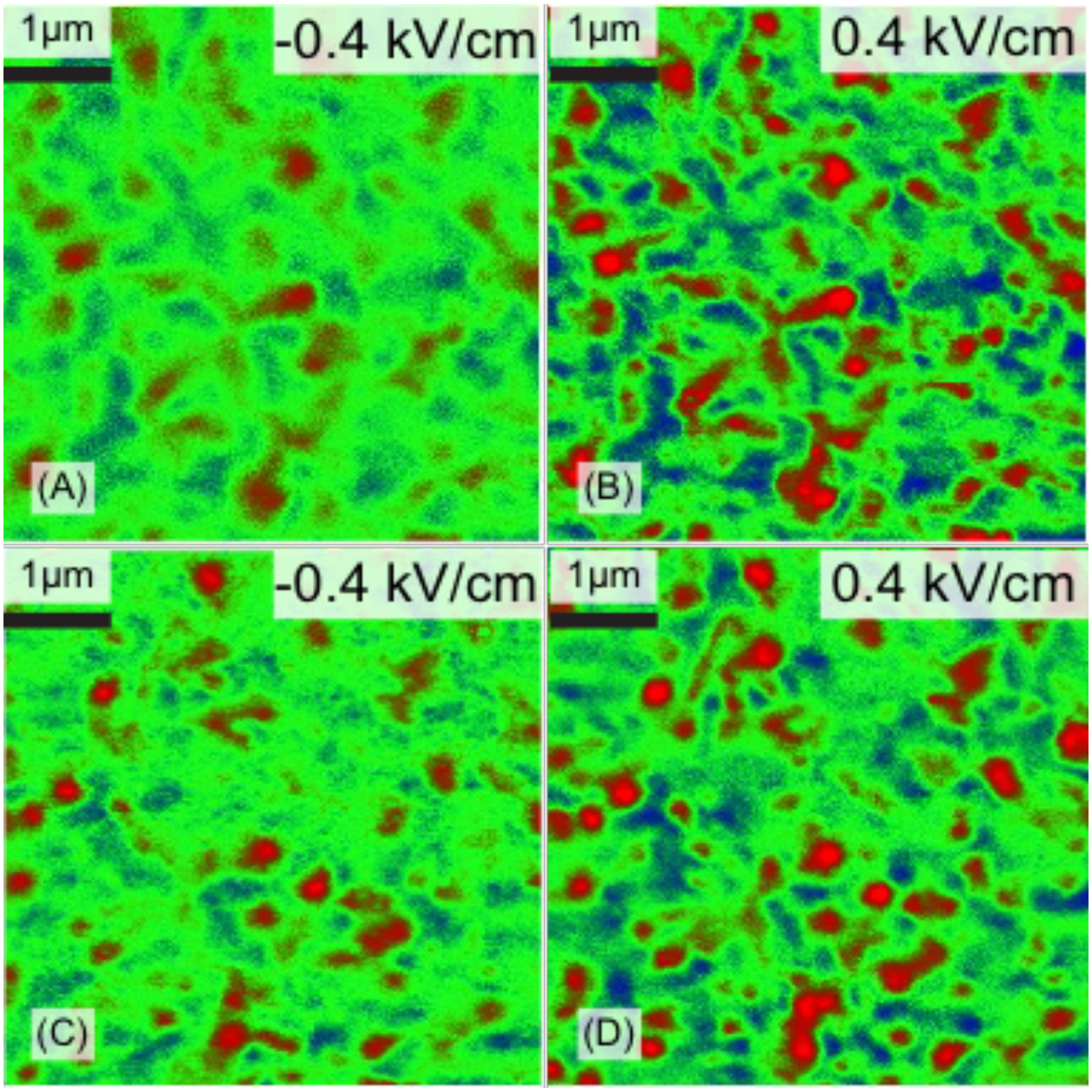

FIG. S7: Magnetic force microscopy of the FeRh/$BaTiO_3$ at 60°C after a DC electric field of ±0.4 kV/cm was applied. Images are labelled sequentially with their corresponding electric field. The magnetic response in (A) and (C) are drastically diminished, when compared with the same region in (B) and (D). The local magnetic memory effect is observed here as the local magnetization is reversibly modified with electric field.
